# Supplementary material for: Elucidating the glycan-binding specificity and structure of Cucumis melo agglutinin, a new R-type lectin
Source: Beilstein J Org Chem. 2024 Feb 19;20:306–20. doi: 10.3762/bjoc.20.31 (PMC10896221; doi:10.3762/bjoc.20.31)
Supplement: File 2 — Additional figures. [file Beilstein_J_Org_Chem-20-306-s002.pdf]

BEILSTEIN JOURNAL OF ORGANIC CHEMISTRY

Supporting Information

for

Elucidating the glycan-binding specificity and structure of *Cucumis melo* agglutinin, a new R-type lectin

Jon Lundstrøm, Emilie Gillon, Valérie Chazalet, Nicole Kerekes, Antonio Di Maio, Ten Feizi, Yan Liu, Annabelle Varrot and Daniel Bojar

Beilstein J. Org. Chem. **2024,** *20,* 306–320. doi:10.3762/bjoc.20.31

Additional figures

License and Terms: This is a supporting information file under the terms of the Creative Commons Attribution License (https://creativecommons.org/licenses/by/4.0). Please note that the reuse, redistribution and reproduction in particular requires that the author(s) and source are credited and that individual graphics may be subject to special legal provisions.
The license is subject to the *Beilstein Journal of Organic Chemistry* terms and conditions: (https://www.beilstein-journals.org/bjoc/terms)

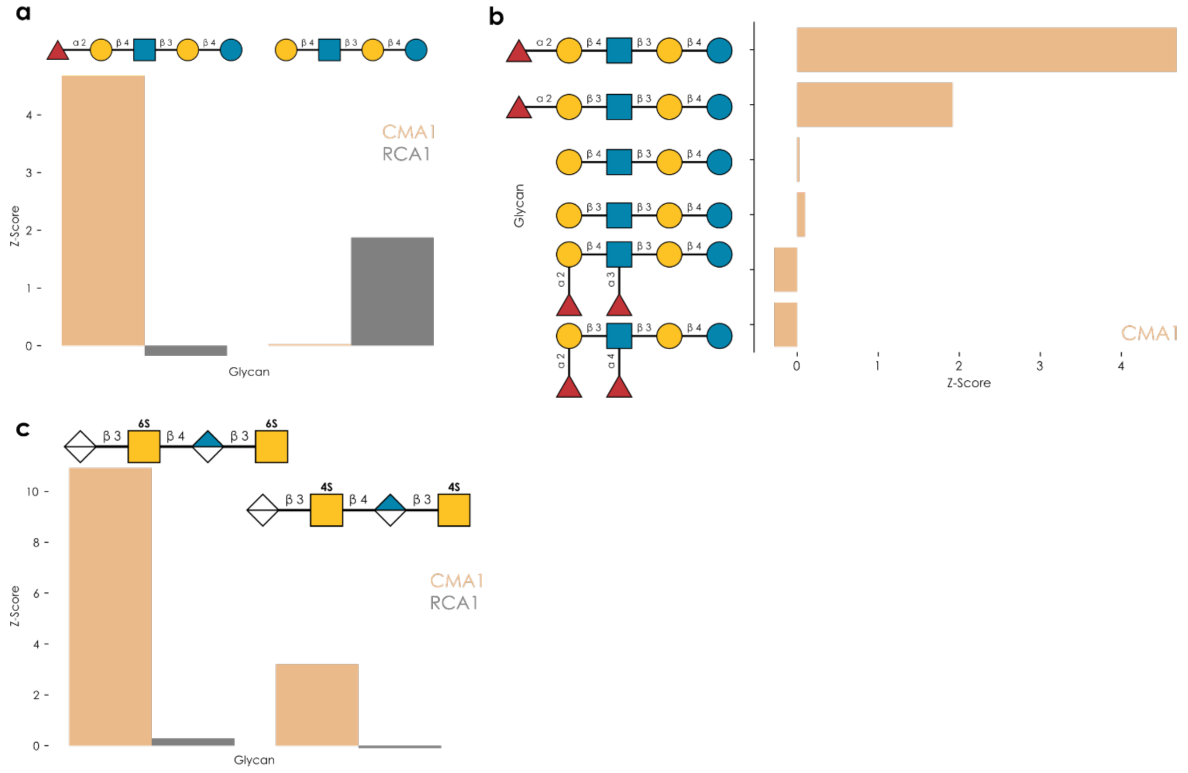

Figure S1: CMA1 strongly prefers C2-substituted galactose for binding. (a–c) For CMA1 and RCA1, we selected *z*-scores from the Imperial College London glycan array data to detail the binding specificity of CMA1. This showed that, while RCA1 requires terminal galactose, CMA1 almost requires the extension of the C2 position of galactose (a). Further, extension of the GlcNAc into a Lewis antigen motif completely abrogated binding of CMA1 to its usual binding motif (b). Example glycosaminoglycans (CS-C and CS-A) indicate strong binding of CMA1, but not RCA1, to sulfated GalNAc.

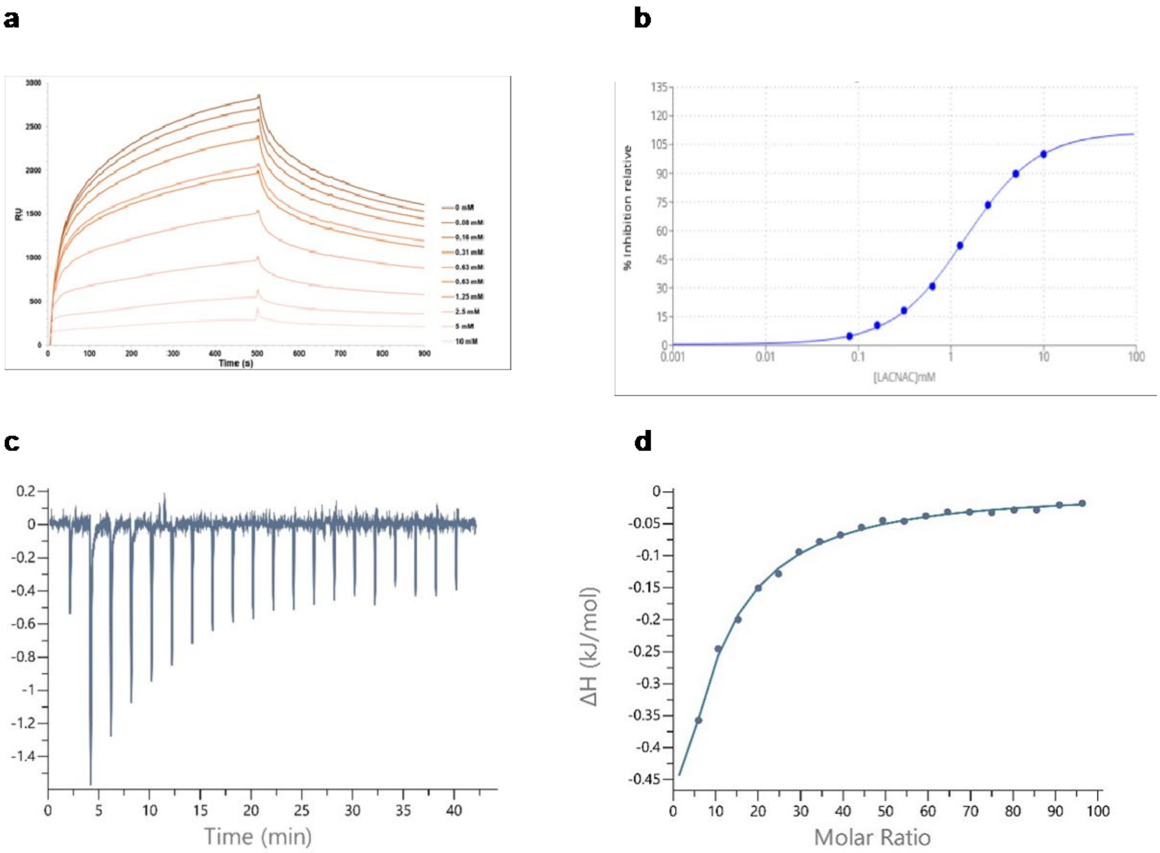

Figure S2: Assessing and quantifying in-solution binding of CMA1. (a, b) SPR analysis of the inhibition of the binding of CMA1 in the presence of LacNAc: multi-cycle sensograms and IC50 analysis. (c, d) ITC analysis of the affinity of CMA1-Nter to GalNAc in solution.

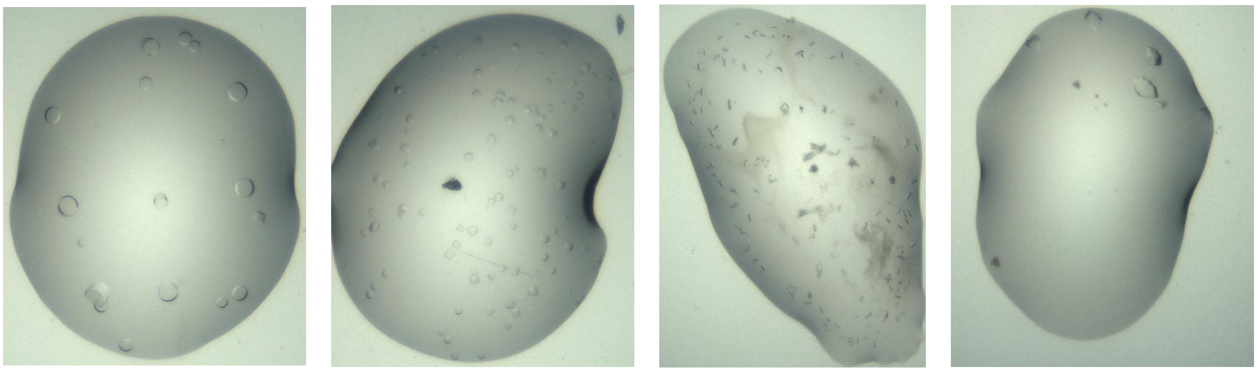

Figure S3: Crystals of the N-terminal domain of CMA1. The crystals were obtained, from left to right, with (i) 1.5 M ammonium sulfate, 0.1 M bis-Tris propane pH 7.0, SaltRX F2; (ii) 4M ammonium acetate, 0.1 M Tris-HCl pH 8.5, SaltRX H10, (iii) 30% PEG 8K, 0.2 M NaCl, 0.1 M imidazole HCl pH 8, Wizard I-II F12, or (iv) 20% PEG 8K, 0.2 M $MgCl_2$, and 0.1 M Tris-HCl pH 8.5, Wizard I+II E3.

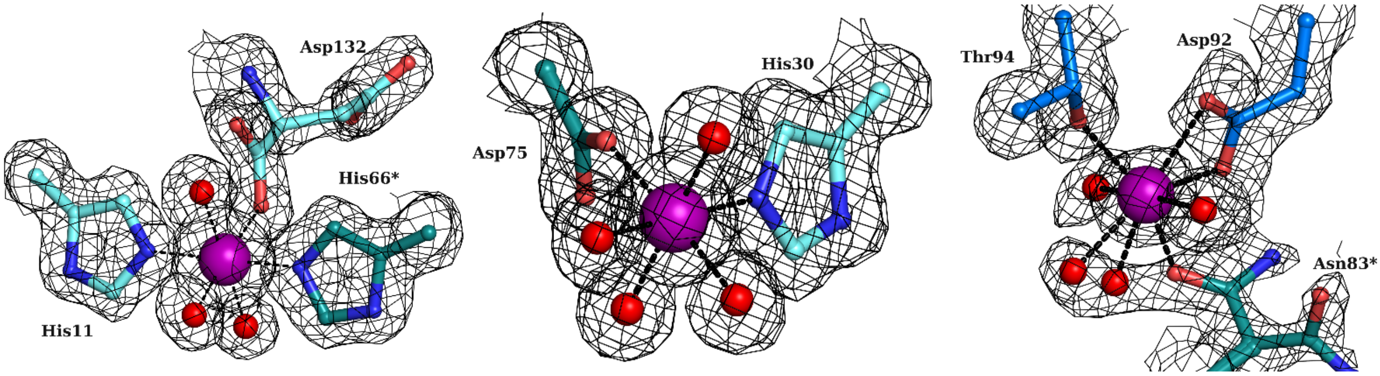

Figure S4: Metal-binding sites in the N-terminal domain of CMA1. Electronic density for cadmium ions binding sites of CMA1 represented at 1 sigma (0.47 e·Å^{-3}). Cadmium is depicted as a purple sphere, waters as red spheres, and amino acids in ball and sticks. Amino acid names with a star represent symmetry related residues.
