# Supplementary material for: Elucidating the glycan-binding specificity and structure of Cucumis melo agglutinin, a new R-type lectin
Source: Beilstein J Org Chem. 2024 Feb 19;20:306–20. doi: 10.3762/bjoc.20.31 (PMC10896221; doi:10.3762/bjoc.20.31)
Supplement: File 3 — Supplementary glycan microarray document (MIRAGE) for the ICL glycan arrays. [file Beilstein_J_Org_Chem-20-306-s003.pdf]

BEILSTEIN JOURNAL OF ORGANIC CHEMISTRY

Supporting Information

for

Elucidating the glycan-binding specificity and structure of *Cucumis melo* agglutinin, a new R-type lectin

Jon Lundstrøm, Emilie Gillon, Valérie Chazalet, Nicole Kerekes, Antonio Di Maio, Ten Feizi, Yan Liu, Annabelle Varrot and Daniel Bojar

Beilstein J. Org. Chem. **2024,** *20,* 306–320. doi:10.3762/bjoc.20.31

Supplementary glycan microarray document (MIRAGE) for the ICL glycan arrays

License and Terms: This is a supporting information file under the terms of the Creative Commons Attribution License (https://creativecommons.org/licenses/by/4.0). Please note that the reuse, redistribution and reproduction in particular requires that the author(s) and source are credited and that individual graphics may be subject to special legal provisions.
The license is subject to the *Beilstein Journal of Organic Chemistry* terms and conditions: (https://www.beilstein-journals.org/bjoc/terms)

Supplementary glycan microarray document based on MIRAGE Glycan Microarray guidelines (doi:10.3762/mirage.3) for the ICL glycan arrays.

Classification	Guidelines
**1. Sample: Glycan Binding Sample**	
Description of Sample	Sample name: Cucumis melo agglutinin (CMA1)<br>Origin: recombinant<br>Method of preparation:<br>Please see the *Experimental* section in the main text.<br>Included for comparison is the biotinylated plant lectin *Ricinus Communis* Agglutinin I (RCA1) from Vector Laboratories.
Sample modifications	Not relevant.
Assay protocol	Microarray analyses were performed essentially as described (Liu et al., Methods Mol. Biol. 2012), for modifications of the protocols please see "Glycan array experiments" under *Experimental* section in the main text.
**2. Glycan Library**	
Glycan description for defined glycans	A screening microarray of sequence-defined lipid-linked glycan probes (neoglycolipids, NGLs). Probe names, glycan structures and lipid tag information of the 866 probes are in **Supplementary Table 2**. These probes are from the collection assembled in the course of research in the Glycosciences Laboratory https://glycosciences.med.ic.ac.uk/glycanLibraryList.html).
Glycan description for undefined glycans	Not relevant.
Glycan modifications	For NGLs, unless otherwise specified these were prepared from reducing oligosaccharides by reductive amination with the amino lipid, 1,2-dihexadecyl-*sn*-glycero-3-phosphoethanolamine [(DHPE) (Chai et al., Methods Enzymol. 2003)]; AO, NGLs prepared from reducing oligosaccharides by oxime ligation with an aminooxy functionalized DHPE [(AOPE) (Liu et al., Chem. Biol. 2007)].<br>For full description on the definition of lipid moieties of the glycan probes please see https://glycosciences.med.ic.ac.uk/docs/lipids.pdf
**3. Printing Surface; e.g., Microarray Slide**	
Description of surface	Nitrocellulose-coated glass microarray slides.
Manufacturer	16-pad UniSart® 3D Microarray Slide from Sartorius (Goettingen, Germany)

Custom preparation of surface	Not relevant.
Non-covalent Immobilisation	The lipid-linked oligosaccharide probes were formulated as liposomes by adding carrier lipids, 1,2-dihexanoyl-*sn*-glycero-3-phosphocholine (DHPC) and cholesterol for arraying and non-covalent immobilization on nitrocellulose-coated glass slides (Liu et al., Methods Mol. Biol. 2012).
**4. Arrayer (Printer)**	
Description of Arrayer	Nano-Plotter 2.1 (GeSiM, Radeberg, Germany).
Dispensing mechanism	Non-contact liquid delivery with four dispensing tips.
Glycan deposition	Approximately 0.33 nl was printed per spot.<br>Lipid-linked glycan probes were printed at 2 and 5 fmol per spot, and polysaccharides at 0.03 and 0.1 ng per spot, all in duplicate.
Printing conditions	The printing solutions were all aqueous based. Printing was performed at ambient temperature and relative humidity of 58%.<br>The ‘liposome’ printing solutions contained 100 pmol/µl of DHPC and cholesterol (both from SIGMA) as lipid carriers in addition to the lipid-linked glycan probes. The concentrations of the lipid-linked glycan probes were 5 and 15 pmol/µl for the 2 and 5 fmol per spot levels, respectively.<br>The printing solutions also contained Cy3 NHS ester (GE Healthcare) at 20 ng/ml (26 fmol/µl) as a marker to monitor the printing process.
**5. Glycan Microarray with “Map”**	
Array layout	Each array slide contained 16-pad subarrays. Each pad was set up for printing 64 probes maximum, each at 2 levels in duplicate (four spots for one probe in a row); up to 256 spots (16x16) in total in each pad.<br>The 866 lipid-linked probes in the screening arrays were printed on multiple subarrays for parallel binding analyses.
Glycan identification and quality control	The quality control of the screening microarrays of sequence-defined glycan probes was carried out with a collection of biotinylated plant lectins including Concanavalin A (ConA), *Aleuria aurantia* lectin (AAL) and wheat germ agglutinin (WGA) (all from Vector Laboratories). The immobilization of the GAG oligosaccharide probes was evaluated with human FGF2, anti-CS and anti-KS antibodies (Wu et al. Mol Cell Proteomics. 2019). These data will be described elsewhere and are available upon request.<br>The glycan probes that may have inaccurate immobilization amounts, as indicated by our quality control experiments, are shaded in grey in Supplementary Table 4.
**6. Detector and Data Processing**	

Scanning hardware	GenePix 4300A (Molecular Devices, UK)
Scanner settings	Scanning resolution: 10 µm / pixel<br>Laser channel: Red (scan wavelength 635 nm)<br>PMT: 350<br>Laser power used for scanning the arrays to achieve maximum signal without spot saturation: 50% for CMA1; 15% for RCA1.
Image analysis software	GenePix® Pro 7 (Molecular Devices)
Data processing	The gpr files were entered into an in-house microarray database using software (designed by Mark Stoll, http://www.beilstein-institut.de/en/publications/proceedings/glyco-2009) for data processing. No particular normalization method or statistical analysis was used for the results of the screening arrays.<br>Data were transformed into z-scores by subtracting the mean value across the array and dividing the results by the standard deviation.
**7. Glycan Microarray Data Presentation**	
Data presentation	The microarray binding results are in **Figure 2,** and **Supplementary Table 2.**
**8. Interpretation and Conclusion from Microarray Data**	
Data interpretation	No software or algorithms were used to interpret processed data.
Conclusions	At the assay condition used, the recombinant CMA1 lectin showed binding showed a broad range of binding activities and bound four types of glycan sequences: galactose (Gal)-terminating, GalNAc-terminating, fucose (Fuc)-terminating and chondroitin sulphate (CS) related sequences. Further details regarding the specificity of the lectin are elaborated upon in the main text.
